# Detection of Epistasis for Flowering Time Using Bayesian Multilocus Estimation in a Barley MAGIC Population

**DOI:** 10.1534/genetics.117.300546

**Published:** 2018-02-25

**Authors:** Boby Mathew, Jens Léon, Wiebke Sannemann, Mikko J. Sillanpää

**Affiliations:** * Institute of Crop Science and Resource Conservation, University of Bonn, 53115, Germany; † Plant Breeding, Martin Luther University of Halle-Wittenberg, 06120, Germany; ‡ Department of Mathematical Sciences, University of Oulu, FIN-90014, Finland; § Biocenter Oulu, University of Oulu, FIN-90014, Finland

**Keywords:** Multiparent Advanced Generation Inter-Cross (MAGIC), multiparental populations, three-way epistatic interactions, sure dependence screening, multilocus association model, multiparental populations, MPP

## Abstract

Gene-by-gene interactions, also known as epistasis, regulate many complex traits in different species. With the availability of low-cost genotyping it is now possible to study epistasis on a genome-wide scale. However, identifying genome-wide epistasis is a high-dimensional multiple regression problem and needs the application of dimensionality reduction techniques. Flowering Time (FT) in crops is a complex trait that is known to be influenced by many interacting genes and pathways in various crops. In this study, we successfully apply Sure Independence Screening (SIS) for dimensionality reduction to identify two-way and three-way epistasis for the FT trait in a Multiparent Advanced Generation Inter-Cross (MAGIC) barley population using the Bayesian multilocus model. The MAGIC barley population was generated from intercrossing among eight parental lines and thus, offered greater genetic diversity to detect higher-order epistatic interactions. Our results suggest that SIS is an efficient dimensionality reduction approach to detect high-order interactions in a Bayesian multilocus model. We also observe that many of our findings (genomic regions with main or higher-order epistatic effects) overlap with known candidate genes that have been already reported in barley and closely related species for the FT trait.

## Abstract (precis)

Flowering time is a well-known complex trait in crops and is influenced by many interacting genes. In this study, Mathew *et al*. identify two-way and....

EPISTASIS can be defined as the presence of genetic interaction between two or more loci in their joint effect on the phenotype. Epistasis is likely to play a crucial role in the genetic variation underlying many complex traits in plants, animals, and humans. Even though the role of epistasis remains controversial, many studies have been devoted to the detection of epistasis in complex traits (*e.g.*, Shimomura *et al.* 2001; Mäki-Tanila and Hill 2014; Huang and Mackay 2016; Paixão and Barton 2016).

Traditionally, quantitative trait loci (QTL) mapping studies use biparental populations to identify QTL specific to the trait of interest. However, the precision of biparental QTL mapping is low due to the limited number of recombination events (Huang *et al.* 2012). Recently, Cavanagh *et al.* (2008) proposed a Multiparent Advanced Generation Inter-Cross (MAGIC) strategy to improve power and precision in QTL mapping. Subsequently, many studies reported QTL for various traits identified using MAGIC populations of *Arabidopsis thaliana* (Kover *et al.* 2009), rice (Bandillo *et al.* 2013), wheat (Huang *et al.* 2012; Mackay *et al.* 2014), and barley (Sannemann *et al.* 2015) for different traits. So far, studies on epistasis from MAGIC populations are rare, despite the obvious benefits provided by multiparental populations (Ehrenreich 2017).

The single-locus model is widely used for association mapping of both quantitative and qualitative traits. However, most complex traits are controlled by multiple genes, and genome-wide association studies (GWAS) using a single-locus model may lead to less statistical power and biased effect estimates. To overcome this, various multilocus methods including Bayesian least absolute shrinkage and selection operator (BLASSO; Yi and Xu 2008), Elastic-Net (Cho *et al.* 2010), empirical Bayes (Leyland and Davies 2005), and variational Bayes (Logsdon *et al.* 2010) methods have been proposed. The general difficulty of multilocus methods is the collinearity (interdependence) of the marker data. These same methods can also be applied to estimate the two-way epistatic effects of QTL if the set of markers is extended to include all possible pairwise interaction terms as pseudomarkers (*e.g.*, Xu 2007; Li and Sillanpää 2012; Kärkkäinen *et al.* 2015). Although many methods are available, two-way epistasis detection in GWAS is still challenging because of the (i) huge number of possible pairwise interaction terms, (ii) small sample sizes, and (iii) the presence of many single-nucleotide polymorphisms (SNPs) with quite small marker effects. Quantifying three-way epistasis is even more challenging due to the computational complexity (screening through all possible combinations) and mathematical challenges (to separate the additive main effects and the higher-order interactions), and the requirement to have enough samples in each subgroup.

Modeling epistasis is a high-dimensional regression problem, and approaches like multifactor-dimensionality reduction (MDR) (Ritchie *et al.* 2001) and sure independence screening (SIS) (Fan and Lv 2008) have been proposed to reduce the dimensionality of the search space in these high-dimensional regression models. The MDR approach is mainly used to detect gene-by-gene interactions in case control studies (*e.g.*, Ritchie *et al.* 2001; Cho *et al.* 2004; Moore 2004). SIS operates by ordering SNPs according to their marginal correlation with the trait and selects a given number of best candidate SNPs. In a recent study, Kärkkäinen *et al.* (2015) used SIS to preselect the variables for the Bayesian LASSO estimation of two-way epistasis in a multilocus association model [see also the approach of Li *et al.* (2014)].

In Bayesian variable selection methods, hyper-parameter selection and study of the sensitivity of results to those choices are needed. To avoid such tedious processes and to make our algorithm more automated, we decided to use the method from Xu (2003) for variable selection. The method proposed by Xu (2003) specifies a strongly informative prior, which shrinks the effects of unimportant SNPs toward zero during estimation and therefore produces a sparse representation of the model. This method is also called “automatic relevance determination,” because it expresses ignorance with respect to scale and it is parameter-free (MacKay *et al.* 1994; Figueiredo 2003; Neal 2012), in addition to also being closely related to relevant vector machines (Tipping 2001).

Flowering Time (FT) is a key complex trait of interest in agronomic crops and many studies have reported pairwise gene-by-gene interactions affecting the FT in different crops (*e.g.*, Caicedo *et al.* 2004; Durand *et al.* 2012; Maurer *et al.* 2015). Unlike offspring from biparental crossing, the MAGIC population is the result of intercrossing among multiple (eight) founder lines and thus offers greater genetic diversity to detect higher-order epistatic interactions. Therefore, in this study, we used the eight-parent MAGIC barley population (Sannemann *et al.* 2015) to report the main, two-way- and three-way-interacting QTL for the FT trait identified using the Bayesian multilocus association model.

## Materials and Methods

In our analysis, we closely follow the method from Kärkkäinen *et al.* (2015), where analysis is done in multiple steps so that residuals obtained from the previous analysis step are taken as phenotypes of the next step. Kärkkäinen *et al.* (2015) performed the dimension-reducing step in the search space of all possible pairwise interaction terms by applying the SIS algorithm, effectively reducing the original 280 million discrete predictors to 5000 important candidates, making the problem more suitable to multilocus modeling. The main differences between Kärkkäinen *et al.* (2015) and our approach here are the following. (i) They used a maximum *a* *posteriori* probability estimation algorithm of extended Bayesian LASSO while we used Bayesian analysis and Markov chain Monte Carlo (MCMC) sampling introduced by Xu (2003) [see also Hoti and Sillanpää (2006) and Bauer *et al.* (2009)]. (ii) Instead of directly using residuals, we ran several MCMC chains and average mean effect coefficients over multiple chains to minimize influence of collinearity between markers in the analysis, before forming residuals. (iii) In addition to the pairwise epistasis analysis (running two rounds of the algorithm), we also examined three-way (higher order) epistasis (running three rounds of the algorithm). (iv) Finally, instead of receiver operating characteristic curves (which do not need any specific method to judge QTL), we applied the following decision rule: the interactions that “popped up” in our analysis needed to correspond to those found in the literature or show similarity after Basic Local Alignment Search Tool (BLAST) search to findings in other flowering plants before we regarded them as real signals (*i.e.*, QTL) [see Wei *et al.* (2014)]. However, note that this rule may cause some bias to the results if it is applied for traits that have not been broadly studied in any species before.

Following the innovation in Kärkkäinen *et al.* (2015), we use the modified version of the SIS method of Fan and Lv (2008), where correlations between a single pseudomarker and the phenotype are computed one at a time and only a few highest ones are stored in the memory. This speeds up the computation process and saves memory because all possible pairwise and three-way pseudomarkers are not retained in the memory.

Here, we shortly describe the model and the algorithm. Let $y_{i}$ for i=1,2..,n represent the phenotypic value of the ith individual in a MAGIC population with *n* observations, then the multilocus association model can be defined as:

$$y_{i}=\beta_{0}+\sum_{j=1}^{p}x_{ij}\beta_{j}+e_{i}.$$ (1)

Here, $\beta_{0}$ is the population mean, *p* is the total number of markers, $x_{ij}$ is the genotypic value of individual *i* at marker *j* coded as 1 for the genotype AA and −1 for the BB, $\beta_{j}$ is QTL effect associated with marker *j*, and $e_{i}$ corresponds to the residual, following a normal distribution as $e_{i}∼N\left(0,\sigma_{e}^{2}\right).$

With genome-wide marker information, the number of markers (*p*) often exceeds the number of observations (*n*). In such cases, Equation 1 can become an oversaturated model and the ordinary least-squares approach will not provide a unique solution. So variable selection and shrinkage estimation are required to obtain a unique solution for Equation 1 and the Bayesian shrinkage approach is one alternative. A common assumption in shrinkage models is that most of the regression coefficients ($\beta_{j}$) have zero values. In order to include pairwise interactions, Equation 1 can be extended to

$$y_{i}=\beta_{0}+\sum_{j=1}^{p}x_{ij}\beta_{j}+\sum_{k<l}^{p}x_{ik}x_{il}\beta_{kl}+e_{i},$$ (2)

where the regression coefficient $\beta_{kl}$ is the pairwise interaction effect of loci *k* and *l*. As the number of loci increases, simultaneous estimation of main effects ($\beta_{j}$) and the interaction effects ($\beta_{kl}$) from Equation 2 becomes computationally challenging. Due to the extremely large parameter space of Equation 2, one needs to apply dimensionality reduction for the variables to make the use of the multilocus epistatic model practical. Therefore, we used SIS, which is based on the marginal correlation with the trait, to select only a subset of variables to be included in the multilocus epistasis association model. For SIS, we first created the pseudomarker for each pair of SNPs and calculated the correlation with the response variable, then we only retained the d highly correlated pseudomarkers for the epistasis search. When variable selection is applied to both the marker main effects and interaction effects ($\beta_{kl}$) in Equation 2, the interaction effects may be masked from the main effects (Sillanpää 2009; Kärkkäinen *et al.* 2015). Therefore, we first estimated the main effects, followed by the interaction effects that were estimated from the residual-outcome analysis (for details, see below).

We also applied SIS to search for three-locus interactions. For that, first we created the pseudomarkers for a set of three SNPs and calculated the correlation with the response variable; then, we retained the t highly correlated pseudomarkers for the three-way epistasis search. To prevent masking of the interaction effect, we estimated the main effects and two-way and three-way interaction (pseudomarker) effects separately using Equation 1.

The procedure for the two- and three-way epistasis searches can be summarized as follows:

1. Estimate the marker main effects $\beta_{j}$ with the multilocus model.
2. Calculate residuals ($E1_{i}$) as $E1_{i}=y_{i}-\sum_{j=1}^{p}X_{ij}\bar{\beta_{j}}.$
3. Use SIS to select the most correlated d pseudomarkers (corresponding to two-way interaction) $X1_{ij}$, to the residual $E1_{i}.$
4. Estimate the two-way interaction effects ($\beta 1_{j}$) with the multilocus model using $E1_{i}$ as the response variable.
5. Calculate the epistasis residuals ($E2_{i}$) as $E2_{i}=E1_{i}-\sum_{j=1}^{d}X1_{ij}\bar{\beta 1_{j}}.$
6. Apply SIS to select the most correlated t pseudomarkers (corresponding to three-way interaction) to the residual $E2_{i}$.
7. Estimate the three-way interaction effects ($\beta 2_{j}$) with the multilocus model using $E2_{i}$ as the response variable.

Here, in steps 2 and 6, the estimates $\bar{\beta_{j}},$ $\bar{\beta 1_{j}}$ were calculated as the average over five different MCMC chains.

Bayesian estimation requires the prior specification for the unknown parameters in the Equation 1. Following Xu (2003), the marker effects were assigned a normal distribution with mean zero and effect-specific variance $\sigma_{j}^{2}.$ For the effect-specific hyper-parameters we assigned Jeffreys’ scale invariant prior, thus, $p\left(\sigma_{j}^{2}\right)\propto 1/\sigma_{j}^{2}$ for j=1,…p. The prior density for the mean $\beta_{0},$ is $p\left(\beta_{0}\right)\propto 1.$ Let $\beta =\left\{\beta_{j}\right\}$ and $\sigma^{2}=\left\{\sigma_{j}^{2}\right\}$ for j=1,2,…p be the unknown model parameters; then, the likelihood of the observation vector *y* is

$$p\left(y|\beta ,\sigma^{2}\right)\propto {\left(\sigma_{0}^{2}\right)}^{-n/2}\times exp(-\frac{1}{2\sigma_{0}^{2}}\sum_{j=1}^{n}{\left(y_{i}-\beta_{0}-\sum_{j=1}^{p}x_{ij}\beta_{j}\right)}^{2}.$$ (3)

By Bayes theorem, the joint posterior distribution of the model parameters is proportional to

$$p\left(\beta ,\sigma^{2}|y\right)\propto p\left(y|\beta ,\sigma^{2}\right)p\left(\beta ,\sigma^{2}\right).$$ (4)

We applied Gibbs sampling (Geman and Geman 1984) to draw samples from the above joint posterior distribution. See Xu (2003) for more details about the implementation of the Gibbs sampling algorithm. Program codes developed during the project are publicly available and listed in Supplemental Material, File S1, File S2, File S3, File S4, and File S5.

### Data set

To validate our approach, we analyzed a barley MAGIC double-haploid (DH) population of 533 lines using the multilocus model for identifying the two- and three-way epistatic interactions. This population was derived from an eight-way cross and the phenotypic data were collected from the research station “Poppelsdorf” of the University of Bonn, Germany. We used the FT phenotype, which was collected during the year 2011, with two replications and we considered the mean over the replications for the analysis. The experiment was arranged in an augmented design and the FT was measured in days until heading [see Sannemann *et al.* (2015) for more details about the experiment]. The population was genotyped using an Illumina 9 k iSelect SNP chip from TraitGenetics GmbH. After discarding the monomorphic markers, ∼3413 SNPs were available for the analysis. 

### Data availability

The data set used in this study can be found as Supplemental Material (File S7 and File S8).

## Results

We estimated the main and interacting QTL in the barley MAGIC DH population using the multilocus model. Of the 3413 SNP markers available for analysis, 1082 SNPs were duplicated (*i.e.*, pairwise marker correlation was nearly 1). During the initial MCMC analysis, we found that if “significant” SNPs are duplicated, Bayesian MCMC-based variable selection will select only one SNP among those duplicated ones. However, in the next MCMC run it may pick another SNP among those duplicated SNPs. High collinearity between markers generally weakens QTL signals by attributing arbitrary parts of every signal to the duplicated marker (*e.g.*, Pasanen *et al.* 2015). Therefore, when we take the average over different MCMC runs there is a possibility that the effect may cancel out and the marker may not appear to be a positive finding. To avoid this ambiguity, we decided to remove those duplicated SNPs and the remaining 2331 markers were used for the final analysis. For the marker effect estimation, we used five different MCMC chains (with different random number generator), each having 50,000 iterations with a burn-in period of 10,000 iterations, and only picked markers that were constantly selected in all chains as QTL. We examined the convergence of the MCMC chain using the trace plots and they showed rapid convergence; therefore, we decided to use 40,000 iterations to obtain the posterior estimates in each case.

### Main effect

FT is a well-studied complex trait and many candidate genes are already known in various plant species. Most of our identified QTL were close to the already reported candidate genes in barley. However, some of the main and interacting QTL regions are not associated with any reported candidate genes in barley. So, we looked into upstream and downstream regions of the putative QTL and performed a BLAST search to identify closest homologous genes for FT in other plant species, and those findings are reported here for the unknown QTL region. The length of the upstream and downstream regions to be studied were decided based on the observed linkage disequilibrium (LD) ($r^{2}>0.8$) around the putative QTL. Main effect QTL for this population were already reported by Sannemann *et al.* (2015) using a single-locus model with binary and haplotype approaches. Unlike the previous study, we used a Bayesian multilocus association model and our results were close to those obtained with the haplotype approach by Sannemann *et al.* (2015). We found that four main effect QTL were common to both studies. In addition, we found evidence in favor of three more main effect QTL. Among these seven identified QTL, four were already known to be involved in FT regulation and reported by many other studies in barley (Wang *et al.* 2010; Maurer *et al.* 2015). The four known regions of major FT genes are: (i) the region ∼19.9 cM on chromosome 2H with the candidate gene pseudoresponse regulator (*PPD-H1*), which provides adaptation to photoperiod in barley (Turner *et al.* 2005); (ii) location 109.20 cM on chromosome 3H with a candidate semidwarf *SDW1* gene (Kuczyńska *et al.* 2013); (iii) region 125.76 cM on 5H with the candidate vernalization (*VRN-H1*) gene (von Zitzewitz *et al.* 2005); and (iv) location 34.34 on 7H with the vernalization (*VRN-H3*) gene (Yan *et al.* 2006).

**Figure 1:**
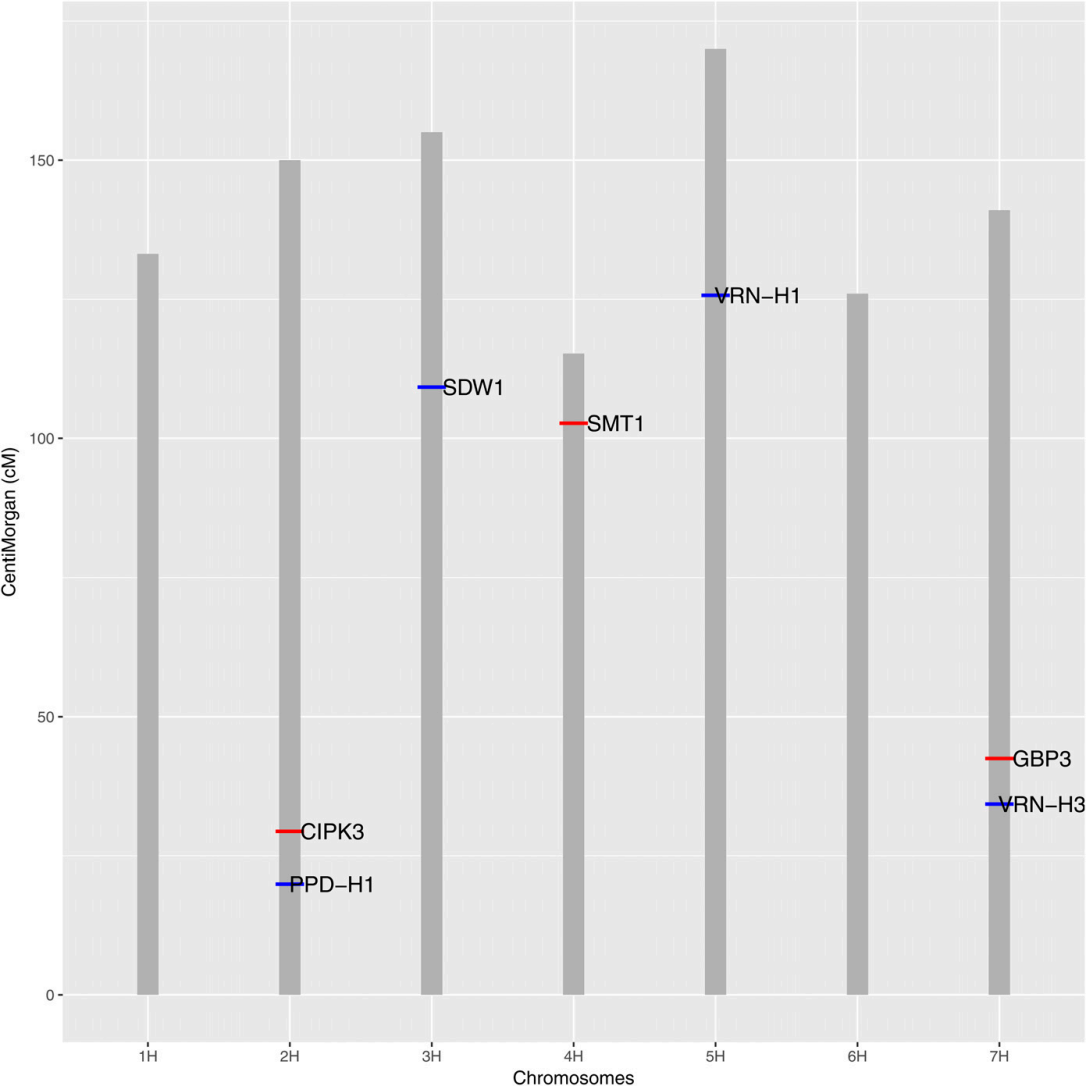
Genes associated with the identified QTL in this study are shown on the corresponding chromosomes. Here, the *x*-axis represents the distance in centiMorgans (cM) and the *y*-axis corresponds to the chromosomes. Blue indicates the QTL already reported by Sannemann *et al.* (2015) and the novel QTL regions found in this study are marked in red.

**Figure 2:**
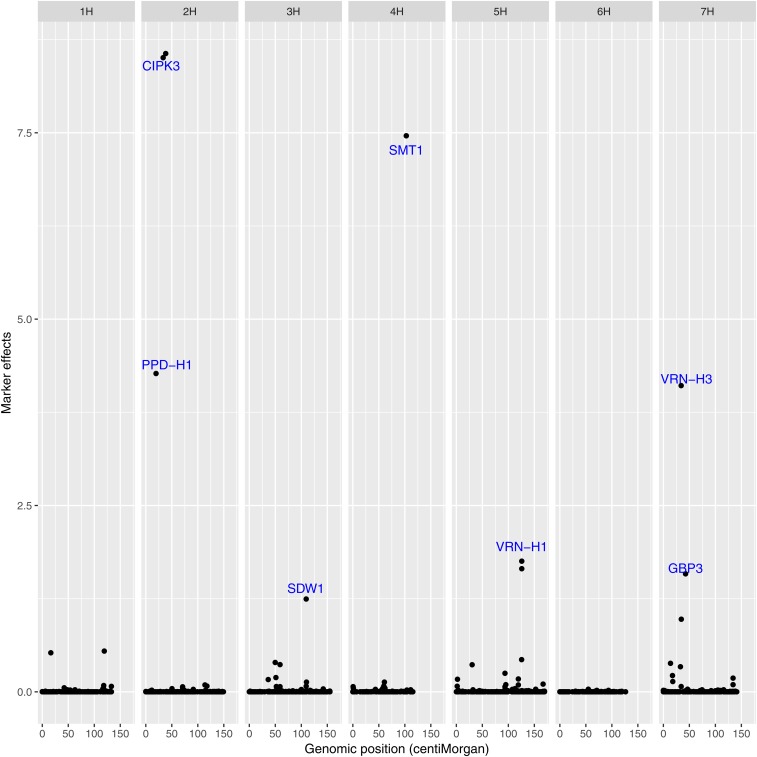
Marker effects estimated in days as posterior means for the flowering time trait with the Bayesian multilocus association model plotted against the corresponding markers in the barley Multiparent Advanced Generation Inter-Cross population.

We also detected two clear QTL signals on chromosome 2H at location 29.01 cM [this region has already been reported by Alqudah *et al.* (2016)] and 33.49 cM. LD analysis for this region showed that these two markers are in high LD, and so we consider this as one QTL region. Since this region has not been previously implicated in FT regulation, we obtained the overlapping protein sequence at 29.01 cM on 2H from Ensembl Plants (www.plants.ensembl.org) for the barley genome and performed a protein BLAST (pBLAST) search against the National Center for Biotechnology Information protein database (https://www.ncbi.nlm.nih.gov/protein/). The pBLAST showed > 92% similarity with the gene “CBL-interacting protein kinase 3 (*CIPK3*)” in various flowering plants. Kim *et al.* (2003) showed that *CIPK3* regulates abscisic acid regulation in *Arabidopsis*. Abscisic acid is known to be involved in variety of physiological processes in plants like seed dormancy (González-García *et al.* 2003), leaf senescence (Zhao *et al.* 2016), and responses to abiotic stresses (Fujii and Zhu 2009). We also performed a pBLAST search as mentioned above for the other two unknown regions, 102.7 cM on 4H and 42.49 cM on 7H, which were found as QTL in our analysis. pBLAST search for the region 102.7 cM on 4H showed high similarity with the gene “Cycloartenol-C-24-methyltransferase (*SMT1*)” in different flowering plants. *SMT1* plays a key role in sterol biosynthesis, and studies have already reported the influence of sterols on plant development (Carland *et al.* 2010), embryogenesis (Clouse 2000), and hormone signaling (Lindsey *et al.* 2003) in *Arabidopsis*. We also calculated the LD for the region between the two QTL on 7H and found that they are not in LD. The associated SNP at 42.49 cM on 7H was located in the gene “*GAMYB*-binding protein (*GBP3*),” and Gubler *et al.* (1995) previously identified gibberellin-specific transcriptional regulator *GAMYB* in barley aleurone cells. Kaneko *et al.* (2004) reported that mutations in the rice *GAMYB* gene retard the growth and development of anthers. Millar and Gubler (2005) reported that *A. thaliana* genes *MYB33* and *MYB65*, which have high sequence similarity to the barley *GAMYB* gene, are regulated by microRNA, and they facilitate anther development. Thus, we believe that this gene is involved in the FT regulation of the MAGIC population. Figure 1 shows the main effect QTL on the corresponding chromosomes. Additionally, we also show the marker effects estimated using a Bayesian multilocus association model in Figure 2, which indicates that the model can clearly separate the QTL signals in the data.

### Two-way epistatic interactions

**Figure 3:**
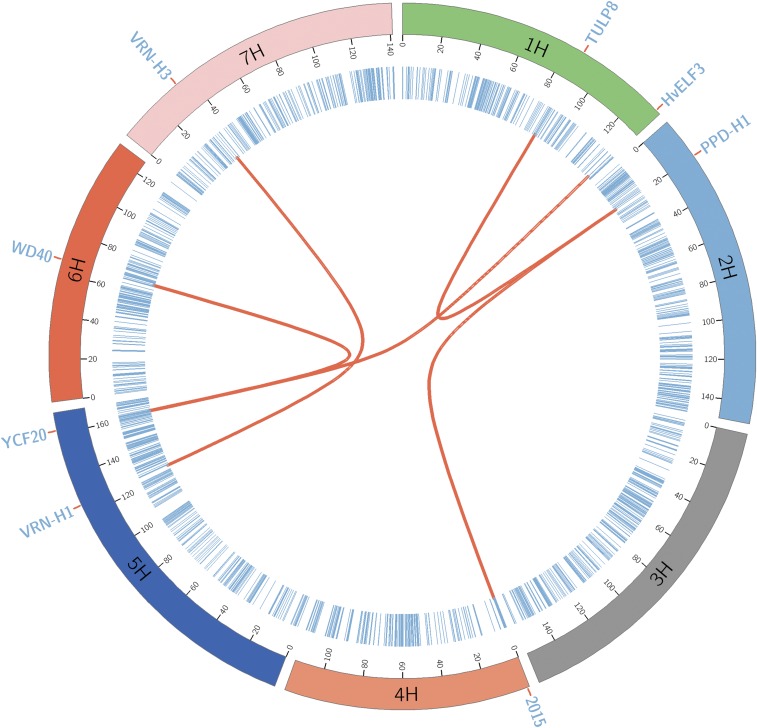
Genetic map of the barley Multiparent Advanced Generation Inter-Cross population with the epistatic interactions. The chromosomes are shown with different colors and the markers in blue lines. The two-way-interacting QTL are connected with red lines and the candidate genes associated with the regions are also shown. Here, “2015” is the region reported by Maurer *et al.* (2015)

To investigate the two-way epistasis, we first applied the SIS and selected 1000 pseudomarkers most correlated with the residuals to estimate the interaction effects with the multilocus model. We found six major two-way-interacting QTL, including the main effect regions on 2H at 29.01 and 33.49 cM, suggesting that these regions are involved in the same interaction. However, due to the high LD between those markers, we consider them to be spurious epistatic signals due to LD rather than any real epistatic finding (*cf*. Wood *et al.* 2014; Kärkkäinen *et al.* 2015). The photoperiod response gene [*PPD-H1* region (19.9 cM on 2H)] was involved in two epistasis interactions, with the region 1.93 cM on 4H and 87.87 cM on H1. Here, the region ∼2 cM on 4H has been already reported by Maurer *et al.* (2015) as a main effect QTL in their study. pBLAST search for the overlapping gene at 87.87 cM on H1 showed high similarity with “Tubby-like F-box protein 8 (*TULP8*),” a member of the TLP gene family, which is composed of 11 members (*AtTLP1-11*) in *Arabidopsis*. The plant-specific transcription factor *LEAFY (LFY)* plays an important role in flower formation in *Arabidopsis* and LFY is a target of *AtTLP8* (William *et al.* 2004; Winter *et al.* 2011). Also, the region ∼161.80 cM on 5H is involved in two interactions, one with the region 130.01 cM on H1 and the other ∼66.78 cM on 6H chromosome. pBLAST search for the overlapping gene on region 161.80 cM on 5H showed > 90% similarity with the gene “ycf20-like protein (*ycf20*)” in other flowering plants. In *Arabidopsis*, it is known that the *ycf20*-like gene affects the thermal dissipation of excess absorbed light (Jung and Niyogi 2010). The region ∼130.01 cM on H1 is known to have the candidate barley clock gene *HvELF3*, which influences the flowering pathway and leads to the early flowering phenotype in barley (Zakhrabekova *et al.* 2012). A pBLAST search for region 66.78 cM on 6H showed high similarity with the gene “tryptophan-aspartic acid (WD) repeat-containing protein” in other species, and WD repeats act as sites for protein–protein interaction in *Arabidopsis* (Van Nocker and Ludwig 2003). QTL regions with the barley vernalization genes *VRN-H1* (125.76 cM on 5H) and *VRN-H3* (34.34 cM on 7H) also showed high signals for two-way epistasis and are known to play a crucial role in FT regulation in barley (Yan *et al.* 2006). Figure 3 represents the genetic map of the major two-way epistatic QTL, which are connected by red lines, and the chromosomes are arranged circularly with the cytobands marked in the inner ring of the plot. The circular plot was created using Circos software (Krzywinski *et al.* 2009). Additionally, for comparison purposes, we also performed a standard a two-dimensional whole-genome scan using PLINK software (Purcell *et al.* 2007). Unlike our multilocus approach, PLINK uses a two-locus model for epistasis searching and will report all the markers that are in high LD with a putative two-way-interacting QTL region as significant markers. Therefore, the PLINK epistasis search provided many significant interaction pairs, and many of these pairs were proximal SNPs that are in high LD to the putative region. The PLINK analysis also provided many significant interactions on the same chromosome, so we removed those SNP pairs from the result. We used a relatively stringent *P*-value of ($10^{-10}$) (Murk and DeWan 2016) to consider the pairs of SNP–SNP interactions as significant and the results are provided in File S6. Four of our five two-way-interacting QTL were also detected by PLINK analysis, except the interaction involving region H1 at 130.01 cM. We think that the observed differences in the identified pairwise interactions are likely due to fundamental differences (in the *Models and Methods*) between the two approaches.

### Three-way epistatic interactions

To detect three-way epistasis, we used a set of 1000 most-correlated pseudomarkers as predictors to explain the two-way corrected residuals in the multilocus model. We also found high collinearity among the pseudomarkers constructed for the three-way epistasis. Therefore, after the Bayesian analysis, we additionally applied an *F*-test to test the significance of the three-way interaction with the null hypothesis ($H_{0}$) that a particular three-way interaction is zero. The *F*-statistic was calculated by comparing model $Y=M_{1}+M_{2}+M_{3}+\left(M_{1}:M_{2}\right)+\left(M_{2}:M_{3}\right)+\left(M_{1}:M_{3}\right)$ against the alternative model $Y=M_{1}+M_{2}+M_{3}+\left(M_{1}:M_{2}\right)+\left(M_{2}:M_{3}\right)+\left(M_{1}:M_{3}\right)+\left(M_{1}:M_{2}:M_{3}\right).$ Here, $M_{i},i=1,2,3$ represents the trio found using the Bayesian multilocus analysis, $M_{i}:M_{j}$ is the two-way interaction term, and $M_{1}:M_{2}:M_{3}$ is the three-way interaction term. We used a significance level of $\text{P}=10^{-5},$ to decide whether a trio of markers is significant and found three major three-way-interacting QTL.

**Figure 4:**
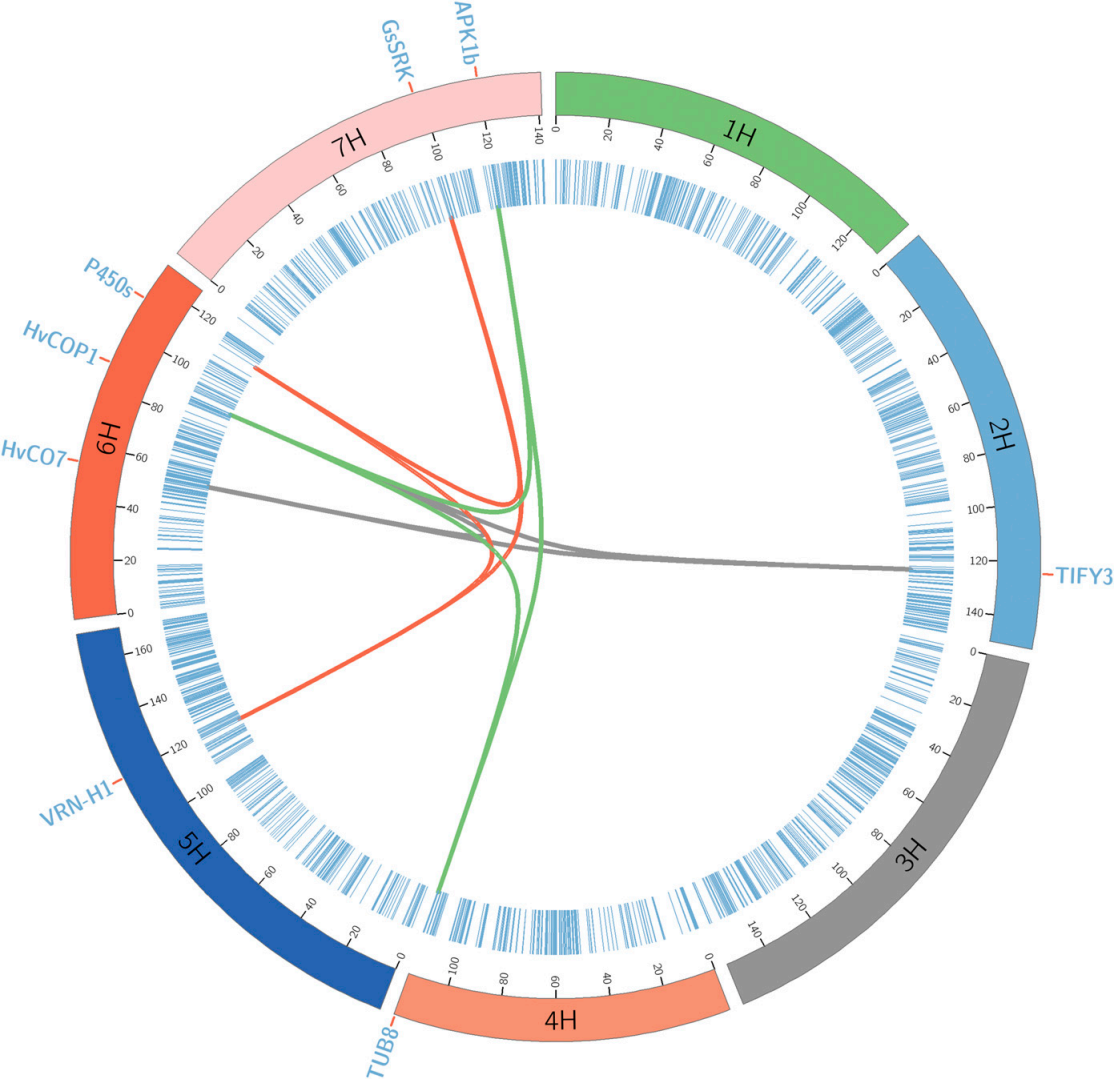
Genetic map of the barley Multiparent Advanced Generation Inter-Cross population with the three-way interactions. The chromosomes are shown with different colors and the markers in blue lines. The trios of three-way-interacting QTL are connected with lines using the same color and the candidate gene associated with the region is also shown.

The regions on 6H at 53.75, and 88.73 and 124.29 cM on 2H, showed the most significant three-way interaction. The region ∼53 cM on 6H is known to have the CO (CONSTANS) gene *HvCO7*, and the CO (CONSTANS) gene has a crucial role in the regulation of flowering by photoperiod in *Arabidopsis* (Griffiths *et al.* 2003), whereas the region 88.73 cM on 6H is known to have the candidate gene CONSTITUTIVELY PHOTOMORPHOGENIC1 (*HvCOP1*), which is required for the UV-B response in *Arabidopsis* (Oravecz *et al.* 2006). However, on 2H at 124.29 cM, we did not find any reported candidate gene, and on pBLAST search we found that this region colocalizes to the gene *TIFY3*. This gene is involved in the jasmonate signaling pathway in different flowering plants and jasmonates play a key role in flower development (Cai *et al.* 2014; Yuan and Zhang 2015; Li *et al.* 2017). In barley, the region 5H (119.8–125.8 cM), spanning *VRN-H1* and PHYTOCHROME C *PHYC*, is known to play a crucial role in FT under long-day photoperiod (Chen *et al.* 2014). In our analysis region, 119 cM on 5H showed significant interaction with the region 113.24 cM on 6H and 97.30 cM on 7H. pBLAST search for the region on 6H (113.24 cM) found that this region colocalizes with the gene Cytochrome P450 (cyt P450) and *cyt P450* enzymes catalyze many reactions in plant metabolism (Bolwell *et al.* 1994; Frank *et al.* 1996). Additionally, *cyt P450* enzymes are involved in the promotion of flowering following vernalization (Burn *et al.* 1993; Dennis *et al.* 1996). The region on 7H at 97.30 cM showed > 95% similarity with gene G-type lectin S-receptor-like serine/threonine protein kinase (*GsSRK*), which is known to be a positive regulator of plant tolerance to salt stress (Sun *et al.* 2013). The region 88.73 cM on 6H with the candidate gene *HvCOP1* was involved in two significant three-way interactions. In the second significant three-way epistasis, it showed interaction with the regions on 7H (119.54 cM) and 4H at 115.22 cM. pBLAST search for the SNP from the region 115.22 cM on 4H showed high similarity with the gene β-tubulin *TUB8*. Previously, Yoshikawa *et al.* (2003) reported anther-specific expression of *TUB8* in rice. Finally, the pBLAST search for the SNP from the region 119.54 cM on 7H showed > 90% similarity with the gene protein kinase 1b (*APK1b*) in *Arabidopsis*. Elhaddad *et al.* (2014) have shown that *APK1b* is predominantly expressed in guard cells and affects light-induced stomatal opening in *Arabidopsis*. Figure 4 represents the genetic map of the major three-way epistatic QTLs and the chromosomes are arranged circularly with the cytobands marked in the inner ring of the plot.

## Discussion

During the past decade, many statistical methods have been developed to identify epistatic interactions in GWAS. However, genome-wide detection of epistasis is still statistically and computationally challenging. In this study, we apply an efficient dimensionality reduction approach to model two- and three-way epistasis in a Bayesian multilocus model. Results from our case study demonstrate that our strategy detects already reported main QTL along with new potential QTL regions. Additionally, we were also able to identify novel two- and three-way-interacting regions involving already reported candidate genes.

We also found that the main difficulty in the use of Bayesian multilocus mapping for epistasis searches in MAGIC populations was the high collinearity (due to the close physical linkage and presence of nearly perfect duplicates) among the markers as well as among pseudomarkers, which created inconsistencies in the results from different MCMC chains. If we presume that a marker (A) is a proxy to strong QTL and that markers A and B are highly similar to each other, then only one of these markers will be selected to the model, and the selection is based on the MCMC starting values. This phenomenon causes unwanted instability in the results from different MCMC chains and the final QTL findings may look ambiguous. On the other hand, in a single-locus model, both markers A and B may easily appear as significant QTL, and having two QTL peaks implies stronger evidence. Additionally, collinearity due to high LD causes problems in SIS by selecting many representative markers close to a putative QTL region. Hence, we emphasize the importance of a quality control step (to remove the duplicates and high-LD markers) before using Bayesian multilocus models with SIS for epistasis searches. In our algorithm, stable performance of the method was also increased by comparing and utilizing estimates from five different MCMC chains. This, combined with carefully made quality control, appears to be helpful and can yield more meaningful results that are replicable.

Computational complexity is another issue that deserves some attention when multilocus association models are applied to main effect and epistasis searches. In this study, the total computation time was ∼90 min for the analysis of the main effects by running 50,000 MCMC iterations in an Intel 8 core processor central processing unit (CPU) with 32 GB random-access memory (RAM). The SIS for the two-way pseudomarkers took ∼5 min on the same computer. Computation time for the two-way epistasis search with 1000 pseudomarkers and the same number of iterations was ∼15 min. However, the time taken to complete the three-way SIS was ∼92 hr, and the estimation of three-way interaction effects with 1000 pseudomarkers took ∼15 min. The three-way SIS with 2331 markers needed to go through all (2331×2330×2329)/3≈4 billion combinations.

Another interesting point we would like to raise is the applicability of the permutation test in multilocus models (Xu 2003). We found that the phenotype permutation test highly depends on the collinearity in the marker data when the multilocus model is applied for association (results are not shown). The upper confidence limit for the permutation test seems to be really high when high between-marker dependency exists. So, as an alternative to permutation, we suggest using the estimates from many MCMC chains with different starting values, and considering the markers that appear in all chains and those previously reported in the literature as the significant ones (Wei *et al.* 2014).

Finally, we note that the marker coding has been identified as having an influence on epistatic QTL findings (He *et al.* 2015; He and Parida 2016; Martini *et al.* 2017). When dealing with MAGIC populations, some researchers defined the numeric codes of genotypes using the identical-by-descent (IBD) approach (Wei and Xu 2016) rather than the identical-by-state (IBS) approach, as in our study. The IBD approach is primarily applied for main effects, because considering epistasis in an eight-parent MAGIC model requires 8 × 8 = 64 possible interaction effects just for a pair of loci. In contrast, using the IBS approach and our assumptions, it is much easier to handle the interactions because only one interaction effect is then needed for a pair of loci. We modeled the interactions between markers as the product of the genotype values and the traditional SNP coding (−1 and 1) that we used might not be able to detect all the epistatic interactions. The SIS approach seeks interactions marginally and therefore one may ask if some interactions are missed, because they do not show sufficiently large signals in SIS analysis. It is true that we may miss interaction signals whose marginal associations are not large enough, but on the other hand, applying SIS for residuals (which loosely do something similar to joint modeling) should partly alleviate this. Also, doing whole analysis multiple times, by varying the number of selected SIS candidates in each analysis, may give some information about sensitivity of the results. However, our results suggest that the dimensionality reduction based on SIS to the residuals is able to detect some of the strong significant higher-order interactions in the real data set. In conclusion, we have illustrated that SIS dimensionality detection can be efficiently combined with Bayesian shrinkage-based variable selection and be successfully applied in real MAGIC populations for three-way epistasis searches, which is otherwise computationally challenging if one goes through all possible combinations by enumeration.

## Supplementary Material

Supplemental material is available online at www.genetics.org/lookup/suppl/doi:10.1534/genetics.117.300546/-/DC1.
